# Function and mechanism of action of the TRPV1 channel in the development of triple-negative breast cancer

**DOI:** 10.3724/abbs.2024068

**Published:** 2024-05-11

**Authors:** Ziling Yan, Haihui Huang, Qianqian Wang, Yanjie Kong, Xia Liu

**Affiliations:** Pathology Department the First Affiliated Hospital of Shenzhen University Shenzhen Second People’s Hospital Shenzhen 518035 China

**Keywords:** ion channel, TRPV1, breast cancer, TNBC

## Abstract

Transient receptor potential channel subfamily vanilloid 1 (TRPV1) is a member of the transient receptor potential family of nonselective cationic transmembrane channel proteins that are involved in the regulation of calcium homeostasis. It is expressed in various tumor types and has been implicated in the regulation of cancer growth, metastasis, apoptosis, and cancer-related pain. TRPV1 is highly expressed in triple-negative breast cancer (TNBC), and both its agonists and antagonists may exert anti-cancer effects. In this review, we provide an overview of the effect of TRPV1 on TNBC development and its influence on immunotherapy in an attempt to facilitate the development of future treatment strategies.

## Introduction

In 2020, for the first time, the incidence and mortality rates of breast cancer in women increased significantly compared to those of women with lung cancer [1]. The latest statistics on the incidence and mortality of new cancers in China show that breast cancer is the most common tumor among females, and its mortality rate is on a continuous rise [2]. Breast cancer is classified into six subtypes based on molecular phenotype and gene expression, namely, luminal-A, luminal-B, HER-2 positive, basal-like (BLBC), normal-like [3] and claudin low [4]. Nearly 70%–80% of BLBC cases are triple-negative breast cancer (TNBC) [5]. Breast cancer is a highly heterogeneous disease with wide variations in its clinical management and prognosis. Existing treatment strategies for breast cancer include surgical resection, chemotherapy, radiotherapy, endocrine therapy, and targeted therapy; however, none of these therapies have adequately addressed the problems of recurrence and metastasis, particularly in TNBC patients. Although PARP inhibitors are indicated for patients with breast cancer carrying BRCA mutations, they do not prolong the overall survival of these patients [6]. In recent years, the immune checkpoint inhibitor (ICI) atezolizumab (which targets PD-L1) in combination with albumin-paclitaxel has shown considerable efficacy against PD-L1-positive infiltrating lymphocytes in TNBC, thereby extending overall patient survival. However, the proportion of the population benefiting from this regimen is small [7], and this regimen was voluntarily withdrawn by Roche in 2021. The anti-PD-1 pembrolizumab combination chemotherapy regimen significantly improves survival to disease progression in patients with locally advanced breast cancer [8] and increases the rate of complete remission in patients receiving neoadjuvant treatment for early-stage TNBC [9]. Due to the lack of effective treatment options, the development of comprehensive therapies and specific therapeutic targets is urgently needed.

Transient receptor potential (TRP) channels are a family of cationic proteins. TRPV5 and TRPV6 are specific calcium channels, while the other members are nonselective [10]. Based on amino acid sequence homology, the mammalian TRP superfamily is divided into six subfamilies: the ankyrin (TRPA), canonical (TRPC), melastatin (TRPM), vanilloid (TRPV), mucolipin (TRPML), and polycytin (TRPP) subfamilies [11]. TRP channel activation is stimulated by various physical or chemical factors both inside and outside the cell, including diglycerides (DAG), inositol trisphosphate (IP3), pH, temperature changes, capsaicin, menthol, and mechanical stimuli [12]. TRP channels are widely distributed in various tissues and play important roles in the regulation of various cellular physiological and pathological functions [13]. Numerous studies have shown that TRP channel expression is important for tumor cell proliferation, migration, and angiogenesis [ 14– 17] . For example, TRPV4, a mechanosensitive ion channel, decreases malignant progression by selectively inhibiting tumor endothelial cell proliferation to reduce tumor angiogenesis [16]. TRPV6 expression increases strongly in high-grade prostate cancer and promotes prostate cancer cell proliferation through a Ca ^2+^/NFAT-dependent pathway [ 18, 19] . Direct targeting or indirect blockade of TRPC5 can effectively overcome resistance to chemotherapy in colorectal cancer (CRC) cells [20]. TRPV6 and TRPM7 are associated with poor survival in patients with breast cancer [ 21, 22] . The TRPC6 channel, which is essential for cell proliferation and cell cycle, is highly expressed in esophageal squamous cell carcinoma (ESCC) and renal cell carcinoma (RCC). TRPC6 channel inhibition induces G2/M phase arrest and suppresses cell proliferation. Therefore, TRPC6 may serve as a novel target for therapeutic intervention in ESCC and RCC [ 23– 25] . Therefore, the TRP channel is considered a diagnostic and therapeutic target for cancer therapy and prognostic prediction.

TRPV1 plays an important role in breast cancer development and treatment; however, a comprehensive review on the relationship between TRPV1 and TNBC is lacking. In this review, we provide an overview of the changes in TRPV1 expression and channel activity associated with TNBC, including changes in clinical prognosis, cell proliferation, migration, invasion, death, and immunotherapy. Elucidating the function and mechanism of action of TRPV1 in TNBC is expected to lay the foundation for future research.

## Functions of TRPV1 in Tumors

The nonselective cation channel TRPV1, also known as capsaicin receptor or vanilloid receptor 1, is a member of the vanilloid subfamily of TRP channels. TRPV1 is expressed in several tissues and can be activated by capsaicin and endogenous cannabinoids such as anandamide (AEA), high temperature (≥43°C), acidic solutions (H ^+^) and inflammatory mediators [ 26– 28] . TRPV1 expression was recently reported to be closely associated with cancer development. It plays a pro-proliferative or pro-apoptotic role by regulating calcium homeostasis and has been implicated in cancer cell proliferation, migration, invasion, and death [ 29– 33] .

Furthermore, TRPV1 is aberrantly expressed in various tumor types [ 34– 41] , and differential TRPV1 expression is often associated with tumor progression and prognosis. Increased TRPV1 expression often indicates poor prognosis. TRPV1 expression correlates with the tumor grade of prostate cancer and increases progressively with Gleason grade [42]. Clinical studies suggest that TRPV1 expression is significantly greater in prostate cancer tissues than in healthy tissues. It can therefore be used as a prognostic indicator and potential therapeutic target for prostate cancer [43]. The TRPV1 agonist capsaicin promotes prostate cancer cell death by phosphorylating LKB1 and activating AMPK, whereas siTRPV1 inhibits LKB1 and AMPK phosphorylation [35]. TRPV1 channel activation inhibits cell proliferation and induces apoptosis, thereby exerting an oncogenic effect. For example, the expression of TRPV1 in hepatocellular and urothelial carcinomas is significantly correlated with a good prognosis. TRPV1 is more strongly expressed in highly differentiated tumors than in healthy cells and poorly differentiated or undifferentiated carcinomas and is related to disease-free survival [ 38, 39] . Similarly, TRPV1 is closely associated with gastric cancer (GC); high TRPV1 expression indicates a good prognosis and is negatively correlated with tumor size, histological grade, and lymph node metastasis in GC. Overexpression of TRPV1 inhibits GC cell proliferation by blocking the G1 phase and attenuating cell migration and invasion [44]. Thus, the role of TRPV1 in different tumors may depend on tumor type and requires further mechanistic studies [45].

## High Expression of TRPV1 in TNBC Is Related to Poor Clinical Prognosis

TRPV1 is a crucial factor in the occurrence and development of breast cancer. Activated TRPV1 induces breast cancer cell apoptosis and inhibits cell proliferation and migration, providing a new approach for the development of treatment strategies [ 46– 49] . The reported expression of TRPV1 varies among different breast cancer subtypes: the highest expression was observed in TNBC, followed by the luminal and basal-like subtypes [ 27, 46, 50] . Weber *et al*. [50] reported that TRPV1 expression is significantly greater in breast cancer tissues than in paracancerous tissues. Lozano *et al*. [51] identified two patterns of immunohistochemical expression of TRPV1 in invasive breast cancer, classical and nonclassical, and the nonclassical pattern is often observed in the more aggressive St. Gallen breast cancer subtype (HER-2+/TNBC). Kaplan-Meier survival curves indicate that patients with the nonclassical TRPV1 expression pattern have lower individual survival rates, and the intracellular localization of TRPV1 has been suggested to be associated with patient survival [51]. These studies show that TRPV1 is associated with a poorer prognosis in TNBC patients. Several studies have evaluated TRPV1 without significant expression differences among breast cancer subtypes [ 52, 53] . Thus, further studies are needed to add more clinical samples for careful evaluation of TRPV1 expression in different subtypes of breast cancer.

## TRPV1 Promotes TNBC Cell Apoptosis through Ca ^2+^ Signaling

**Figure 1:**
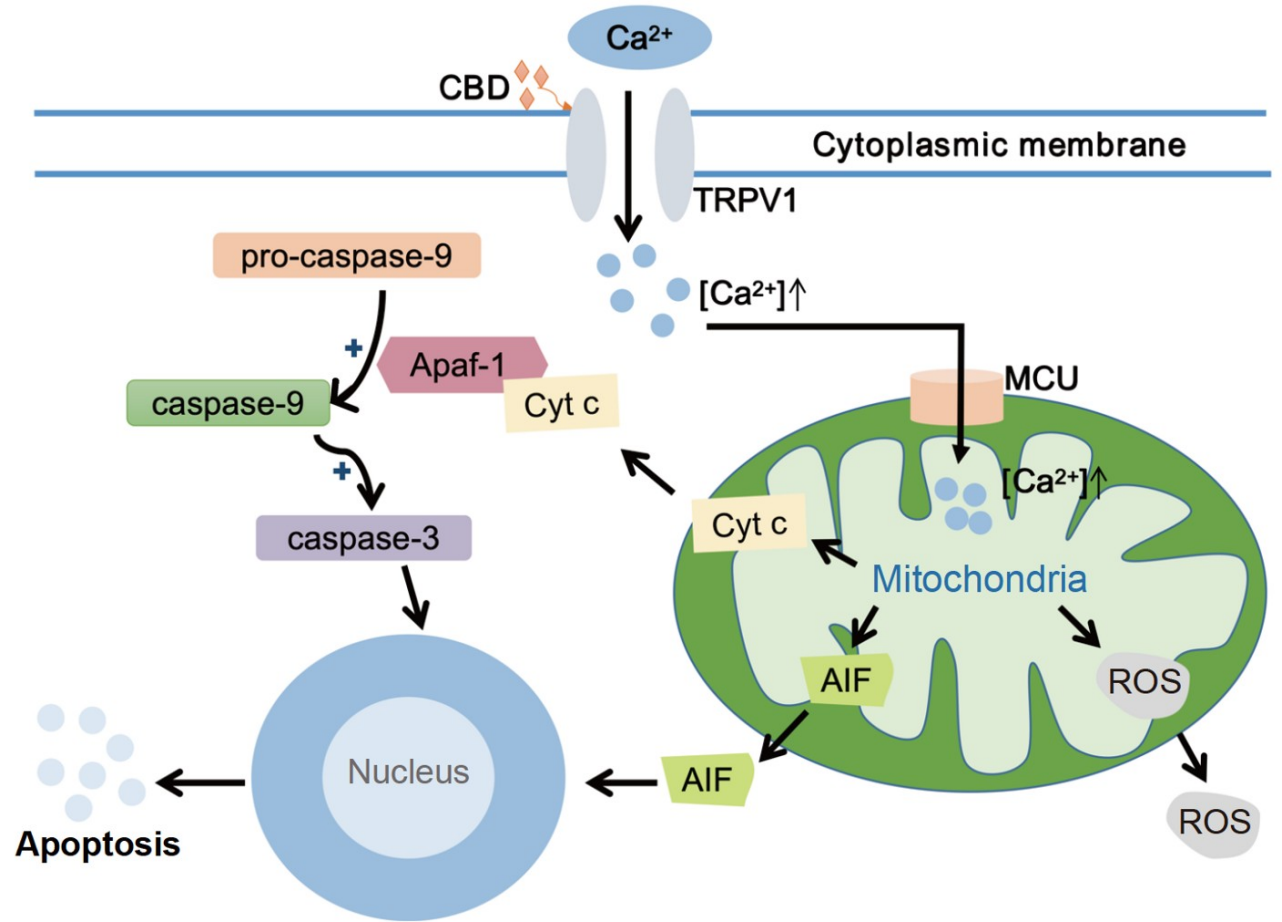
Figure 1 TRPV1-mediated apoptotic cell apoptosis process The TRPV1 agonist receptor cannabidiol (CBD) activates TRPV1 channels, causing extracellular calcium ions to flow inward. Increased intracellular Ca 2+ level induce mitochondrial calcium uniporter transfer, which causes Ca 2+ to enter the mitochondria and release cytochrome C (Cyt c), reactive oxygen species (ROS), and apoptosis-inducing factors (AIFs). AIFs translocate directly to the nucleus, bind to DNA, and induce DNA breakage and condensation. The released Cyt c activates and binds to apoptotic protease-activating factor-1 (Apaf-1) to form a large complex known as apoptotic vesicles, causing it to activate pro-caspase-9, which triggers caspase-9 and caspase-3 activation, ultimately leading to apoptosis. Intracellular ROS supports the activation of p38 MAPKs, thereby promoting ER stress in the cytoplasm.

Ca ^2+^ signaling is associated with breast cancer cell proliferation and migration, and persistent excess intracellular Ca ^2+^ induces breast cancer cell apoptosis [54]. Several TRPV1 agonists promote TNBC cell apoptosis by activating Ca ^2+^ channels [45]; for instance, cannabidiol-activated TRPV1 induces MDA-MB-231 cell apoptosis [55], and capsaicin promotes SUM149PT cell death by stimulating TRPV1 channels [50]. These phenomena may be related to TRPV1 being a calcium-permeable channel and its involvement in intracellular calcium signaling [54]. The proapoptotic effect of TRPV1 involves mitochondrial dysfunction, ROS production, and ER oxidative stress [ 56– 58] . The agonist receptor CBD binds to TRPV1 and activates it, leading to an increase in intracellular Ca ^2+^ level [55] and inducing mitochondrial calcium uniporter transfer, which causes Ca ^2+^ to enter the mitochondria and release cytochrome C, ROS [59], and apoptosis-inducing factor, which triggers caspase-9 and caspase-3 activation ( Figure 1) [ 60– 62] . High amounts of ROS upregulate p38, and MAPKs contribute to ER stress in the cytosol [ 63, 64] . These studies have focused mainly on *in vitro* models, and the role of the TRPV1 channel *in vivo* remains to be elucidated.

## The TRPV1 Agonist Capsaicin Inhibits TNBC

Capsaicin, a primary component of chili peppers and a highly pungent alkaloid of vanillin [65], is also known to be a TRPV1 agonist that has various pharmacological effects, including analgesic [66], antiobesity [67], anti-inflammatory [68], antioxidant [69] and antitumor effects [70]. Capsaicin has been shown to have an antitumor effect on various types of cancer, such as colon adenocarcinoma [71], glioma [37], gastric cancer [72], lung cancer [73], bladder cancer [39], and breast cancer [74]. The antitumor mechanisms are mainly related to its antiproliferative, apoptosis-inducing, antiangiogenic and antimetastatic effects [ 71– 74] . Although some *in vivo* studies using animal models support the antitumorigenic activity of capsaicin, it is possible that capsaicin may act as a carcinogen [ 75, 76] . In addition, the biological function of capsaicin is strongly influenced by its concentration. It has been concluded that the TRPV1 agonist concentration and exposure duration affect the level of inwards calcium flow, which in turn affects TNBC cell invasion and migration [45]. Therefore, we suggest that the dual function of TRPV1 in TNBC is dependent on the dose of capsaicin. In MDA-MB-231 cells, low concentrations of capsaicin (10, 50, and 100 μM) did not have a significant impact on cell viability, while capsaicin at a concentration over 200 μM significantly decreased cell viability. In contrast, compared with 0 μM capsaicin, 10 μM capsaicin inhibited cell migration. The mechanism may rely on capsaicin reducing the protein expression levels of CDK8, Wnt and β-catenin, and further inhibiting CDK8/Wnt/β-catenin signaling to suppress breast cancer cell migration [ 77, 78] . The dose of capsaicin used in these studies is controversial, and further studies on the toxicity and safety of capsaicin are still needed to determine its dosage and usage in clinical applications.

Capsaicin also inhibits TNBC cell migration and invasion through TRPV1-independent channels [79]. Capsaicin inhibits TNBC growth and metastasis by regulating several signaling pathways, such as the NF-κB, MAPK, PI3K/Akt [80] and STAT3 [81] pathways. Additionally, studies have demonstrated that capsaicin can increase the sensitivity and improve the efficacy of TNBC chemotherapy [82]. These studies demonstrated that capsaicin has potential in TNBC treatment. However, whether capsaicin exerts anticancer effects on TNBC through TRPV1 channel activation and how it exerts its mechanism of action through the TRPV1 channel require further in-depth studies and validation.

## Potential Role of TRPV1 Channels in TNBC Immunotherapy

A KEYNOTE-355 phase III randomized controlled study [83] and treatment with an anti-PD-1 agent and nab-paclitaxel determined that TNBC is a significant immunogenic subtype of breast cancer [84]. Activated TRPV1 channels influence immune cell activity and cytokine secretion [85], including dendritic cells (DCs) [86], T-lymphocytes [ 85, 87] and macrophages [88]. TRPV1 activation promotes the proliferation of tumor infiltrating lymphocytes (TILs) and the secretion of immune factors, enhancing their ability to kill tumor cells [ 45, 89] . Furthermore, in tumor tissue, TRPV1 regulates the infiltration and migration of T cells. Capsaicin-activated TRPV1 indirectly induces T-cell proliferation by inducing DC maturation [90], and this strategy is promising for generating relevant vaccines [91]. Warm physical stimulation was found to convert an immunosuppressive state in the tumor microenvironment to an immunoreactive state, thus increasing the receptive of tumor cells to immune checkpoint inhibitor therapy [92]. TRPV1 channels can be activated by heat (≥43°C) and have the potential to induce TME transformation by activating calcium signaling in immune cells and triggering increased secretion of immune factors [29]. Ngo *et al*. [93] delivered capsaicin and ICI (BMS202) into the glutathione-rich TME simultaneously using nanotechnology. The release of capsaicin-activated TRPV1 in the TME enhanced the expression of PD-L1 on the surface of tumor cells and promoted the recruitment of T cells into the TME, increasing immunoreactivity. Simultaneously, BMS202 inhibited immune checkpoints on both tumor cells and T cells, activating the recruited T cells and resulting in the elimination of tumor cells [ 93, 94] . In contrast, nanoparticle-mediated TRPV1 channel blockade in combination with hyperthermia improved heat immunotherapy efficacy in TNBC, and researchers have employed nanoparticle-mediated TRPV1 blockade to inhibit HSP70 expression in TNBC by suppressing HSF1. This combined approach significantly inhibited primary, metastatic, and recurrent TNBC tumor growth [ 95, 96] . By activating or inhibiting TRPV1 channels, the function and infiltration capacity of TILs can be regulated, thereby improving the efficacy of immunotherapy. This strategy may help to overcome tumor impediments to immune escape and improve patient survival and therapeutic efficacy. Therefore, in-depth studies on the interaction between TRPV1 channels and TILs are expected to provide new ideas and strategies for the development of TNBC immunotherapy.

## Conclusion and Prospect

Based on the reviewed evidence, the following conclusions can be drawn. TRPV1 is highly expressed in TNBC. TRPV1 is mainly associated with TNBC cell death through the regulation of calcium homeostasis. The proapoptotic effect of TRPV1 in TNBC is mainly related to mitochondrial dysfunction, ROS production, and ER oxidative stress. Finally, we discussed whether TRPV1 induces antitumor immune responses through specific pathways in TNBC immunotherapy. For instance, activation of TRPV1 channels can influence immune cell activity and cytokine secretion. TRPV1 channels regulate T-cell infiltration and migration in tumor tissues, thereby enhancing their ability to attack tumors. Nanoparticle-mediated TRPV1 channeling can be combined with thermotherapy or ICIs to exert a therapeutic effect on TNBC. Therefore, TRPV1 activation may be a potential therapeutic target for TNBC. To date, most of the evidence has been gathered from *in vitro* models, and the role of the TRPV1 channel *in vivo* remains to be elucidated. Therefore, further studies are needed to explore the direct mechanism of action of TRPV1 in TNBC.
